# C_60_ Fullerene Reduces 3-Nitropropionic Acid-Induced Oxidative Stress Disorders and Mitochondrial Dysfunction in Rats by Modulation of p53, Bcl-2 and Nrf2 Targeted Proteins

**DOI:** 10.3390/ijms22115444

**Published:** 2021-05-21

**Authors:** Olga O. Gonchar, Andriy V. Maznychenko, Olena M. Klyuchko, Iryna M. Mankovska, Kamila Butowska, Agnieszka Borowik, Jacek Piosik, Inna Sokolowska

**Affiliations:** 1 Department of Hypoxic States and Department of Movements Physiology, Bogomoletz Institute of Physiology, Bogomoletz Str. 4, 01024 Kyiv, Ukraine; olga.gonchar@i.ua (O.O.G.); mankovsk@biph.kiev.ua (I.M.M.); 2 Department of Physical Education, Gdansk University of Physical Education and Sport, Kazimierza Gorskiego Str. 1, 80-336 Gdansk, Poland; inna.sokolowska@awf.gda.pl; 3 Department of Electronics, National Aviation University, L. Huzar Ave. 1, 03058 Kyiv, Ukraine; kelenaXX@nau.edu.ua; 4 Laboratory of Biophysics, Intercollegiate Faculty of Biotechnology UG-MUG, Abrahama 58, 80-307 Gdansk, Poland; kamila.butowska@phdstud.ug.edu.pl (K.B.); agnieszka.borowik@phdstud.ug.edu.pl (A.B.); jacek.piosik@biotech.ug.edu.pl (J.P.)

**Keywords:** C_60_ fullerene, 3-nitropropionic acid, mitochondrial dysfunction, oxidative stress, Nrf2/ARE, p53, glutathione system, MnSOD

## Abstract

C_60_ fullerene as a potent free radical scavenger and antioxidant could be a beneficial means for neurodegenerative disease prevention or cure. The aim of the study was to define the effects of C_60_ administration on mitochondrial dysfunction and oxidative stress disorders in a 3-nitropropionic acid (3-NPA)-induced rat model of Huntington’s disease. Animals received 3-NPA (30 mg/kg i.p.) once a day for 3 consecutive days. C_60_ was applied at a dose of 0.5 mg/kg of body weight, i.p. daily over 5 days before (C_60_ pre-treatment) and after 3-NPA exposure (C_60_ post-treatment). Oxidative stress biomarkers, the activity of respiratory chain enzymes, the level of antioxidant defense, and pro- and antiapoptotic markers were analyzed in the brain and skeletal muscle mitochondria. The nuclear and cytosol Nrf2 protein expression, protein level of MnSOD, γ-glutamate-cysteine ligase (γ-GCLC), and glutathione-S-transferase (GSTP) as Nrf2 targets were evaluated. Our results indicated that C_60_ can prevent 3-NPA-induced mitochondrial dysfunction through the restoring of mitochondrial complexes’ enzyme activity, ROS scavenging, modulating of pro/antioxidant balance and GSH/GSSG ratio, as well as inhibition of mitochondria-dependent apoptosis through the limitation of p53 mitochondrial translocation and increase in Bcl-2 protein expression. C_60_ improved mitochondrial protection by strengthening the endogenous glutathione system via glutathione biosynthesis by up-regulating Nrf2 nuclear accumulation as well as GCLC and GSTP protein level.

## 1. Introduction

Mitochondria are key regulators of cell functions and cell survival, therefore any changes in mitochondrial energy metabolism, impaired calcium buffering, and increased generation of reactive oxygen species (ROS) can cause mitochondrial dysfunction and be a trigger for a variety of neurological pathologies [1].

Huntington’s disease (HD) is a progressive and fatal neurodegenerative disorder, characterized by the clinical triad: Movement disorder, dementia, and psychiatric disturbance due to striatal-specific neuronal degeneration [2,3]. The mechanism responsible for neuronal death at HD still remains unknown. However, it was found that abnormal aggregation of mutant huntingtin (mHtt) proteins could cause toxic effects in neurons, followed by a cascade of pathogenic mechanisms associated with bioenergetic defects and subsequent excitotoxicity, mitochondrial dysfunction, oxidative stress, transcriptional alterations, and apoptosis [3,4]. mHtt causes lesions not only in specific brain areas, but also in peripheral tissues like skeletal muscles, kidney, heart, and liver, where mHtt abundance is the same as in the brain [5,6].

Mitochondrial toxin 3-nitropropionic acid, which selectively inhibits complex II of the electron transport chain (ETC), produces clinical and pathologic manifestations of disorders that look like HD symptoms in rodents, primates, and humans [7]. It was registered that 3-NPA easily penetrates the blood–brain barrier and blocks electrons’ transport in ETC, followed by the uncoupling of oxidative phosphorylation, and an energy deficit in the brain [8,9]. Such metabolic disorders cause excessive ROS production, including the production of superoxide radicals, hydrogen peroxides, hydroxyl radicals, and peroxynitrites, and thereby induce oxidative stress, which further leads to damages in lipids, DNAs, and proteins [8,9,10,11].

Studies from some laboratories demonstrated that 3-NPA significantly elevated mitochondrial prooxidant status through the downregulation of antioxidative enzymes, glutathione (GSH) depletion, and the disruption of redox state [10,12]. The removal of free radicals in biological systems is achieved through enzymatic and non-enzymatic antioxidants. [13,14]. GSH plays many important functions in the cell: Maintaining redox homeostasis, free radicals and electrophilic intermediates scavenging, conjugation/detoxification reactions, apoptosis, gene expression, and cell signaling [15]. Matrix GSH redox cycle in coordination with MnSOD-mediated scavenging of the superoxide anion is crucial for preventing excessive ROS accumulation in mitochondria [13,14]. Deficits of GSH and GSH-related enzymes are associated with several neuropsychiatric and neurodegenerative disorders; they often occur earlier than other pathological manifestations of the disease [3,4,11,12]. Therefore, the search for substances influencing GSH synthesis would be a promising approach for neurodegenerative disease therapy.

The endogenous antioxidant response pathway protects the cells from oxidative stress by increasing the expression of cytoprotective enzymes that can scavenge free radicals and reduce the risk of cellular damage caused by ROS [14,15]. The transcription factor Nrf2 (nuclear factor erythroid 2 p45-related factors 2) regulates this pathway via binding to antioxidant response elements (AREs) in the promoter regions of antioxidants [16,17]. Nrf2 controls both the basal and inducible expressions of genes, encoding heavy and light chains of γ-GCL, one of the important enzymes in GSH biosynthesis, as well as other GSH-related enzymes, and thereby it takes part in GSH recycling regulation [18].

Mitochondrial ROS generation and antioxidant capacity are potential points of application for the correction of oxidative stress-induced disorders, including mitochondrial dysfunctional states by pharmacological and molecular means [3,11].

C_60_ fullerene is known as a unique class of carbon allotropes that have conjugated carbon–carbon double bonds, all of which react easily with radical species. Due to its structure, C_60_ can neutralize oxygen free radicals and mimic SOD activity [19,20]. This makes these compounds attractive for biological and medical usage at the prevention of oxidative stress-induced disorders, which underlie many pathological states [21,22,23]. Numerous C_60_ positive effects were determined mainly by the antioxidant capacity of C_60_, which was several hundred times higher than that of other antioxidants [21,22,23,24,25]. Recently, water-soluble fullerenes attracted great attention in neurosciences [26,27,28]. The brain contains many different unsaturated fatty acids, and it also has limited ability to regenerate damaged tissues. All of this makes this organ very sensitive to oxidative damage caused by free radicals [8]. It was shown that fullerene inhibits the LPO chain reactions, glutamate-receptor-mediated excitotoxicity, apoptotic cell death, and in that way demonstrate effective neuroprotection in tissue and cell culture models of neurodegenerative diseases, including Parkinson’s disease [27,28,29,30,31]. However, despite the convincing pieces of evidence describing the neuroprotective properties of water-soluble fullerene and its derivatives, there is only limited information about the mechanisms of their action in ROS-related degenerative disorders like HD. Hence, the therapeutic potential of fullerenes in the treatment of neurologic diseases requires further investigations.

The present study was carried out to elucidate the effect of C_60_ as an antioxidant in weakening oxidative stress, mitochondrial dysfunction, and apoptosis caused by administration of the 3-NPA at a high dose. Many studies have demonstrated that oxidative stress influences profoundly organs with high levels of metabolic activity and ATP utilization such as the brain and skeletal muscle [3,6]. It was well documented that myoblasts from presymptomatic and symptomatic HD individuals show accumulation of intracellular mHtt protein aggregates, impairment of energy metabolism, transcriptional deregulation, and programmed cell death [2,5,32]. In addition, patients with HD and animals in a 3-NPA-induced HD rodent model suffered from weight loss, wasting of skeletal muscle, increased energy expenditure, and disorders in locomotor activity [5,6,33]. Therefore, we investigated 3-NPA-induced oxidative damage of a brain, as well as skeletal muscle, as an important stress-sensitive peripheral organ.

It is well known that nuclear protein p53 functions as a transcription factor for target genes regulating apoptosis, cell cycle, cell respiration, and energy metabolism [34,35]. P53 controls the cell destiny through several mechanisms, depending on stress magnitude. High levels of ROS cause phosphorylation and stabilization of the p53 protein, which often exhibits a pro-apoptotic function under such circumstances. At the same time, a low level of stress stimulates p53 to upregulate the expression of genes encoding ROS-scavenging enzymes [34,35]. Alternatively, p53 could be translocated into mitochondria to regulate apoptosis and oxidative stress through the transcription-independent pathway [36]. The multiple roles of p53 in ROS homeostasis [34,37] and the beneficial C_60_ anti-apoptotic effect [22,30] permitted us to suppose that C_60_ administration would regulate p53 subcellular distribution and influence the cross-talk homeostasis between mitochondrial ROS and p53 activity, thereby potentially delaying 3-NPA-induced mitochondrial dysfunction and apoptosis. Thus, there is a reason for the investigation of p53 accumulation in mitochondria and the protein level of Bcl-2 as a well-known anti-apoptotic agent. In our and other earlier studies, it was demonstrated that C_60_ enhances endogenous phase 2 antioxidant enzymes via Nrf2/ARE-dependent pathways [23,30]. To determine the potential mechanisms of C_60_-mediated antioxidant protection against oxidative stress evoked by 3-NPA exposure, we estimated protein expression of Nrf2 in the nuclear and cytosol fractions, as well as protein level of the MnSOD and GSH-related enzymes as Nrf2 downstream targets. 

## 2. Results

### 2.1. Body Weight Changes

**Figure 1:**
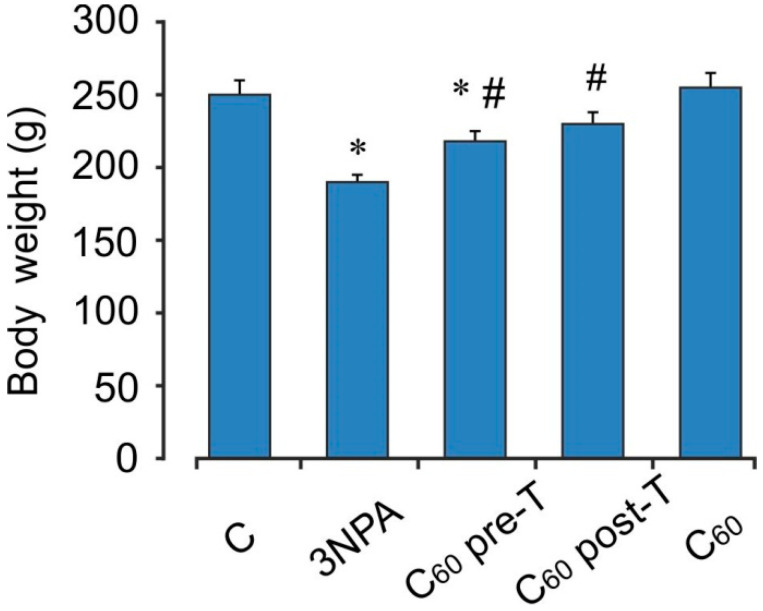
Effect of C_60_ and 3-NPA administration on body weight rats. Values are mean ± SEM (*n* = 10). The data were analyzed for statistical significance using ANOVA followed by the Bonferroni post hoc test. (C)—control; 3NPA—3-nitropropionic acid treated rats; (C_60_ pre-T)—C_60_ administration in pre-treatment regimen; (C_60_ post-T)—C_60_ administration in post-treatment regimen; (C_60_)—C_60_ administration alone. * *p* < 0.05 vs. the control group; ^#^ *p* < 0.05 vs. the 3-NPA treated rats.

3-NPA treatment caused significant decrease in body weight on the last day of the experiment by 24% as compared to the vehicle-treated group (control) (*p* < 0.05). C_60_ administration in pre- and post-treatment regimen significantly reversed the 3-NPA-induced decrease in body weight and the increase in body weights was found to be by 16 and 21%, respectively (*p* < 0.05) (Figure 1).

### 2.2. Neurological Scoring

The neurological scoring, based on movement analysis is depicted in Table 1. Administration of 3-NPA alone resulted in motor abnormalities, and none of these rats showed normal behavior or general slowness. Motor in-coordination and marked gait abnormalities were observed in four animals, hind limb paralysis in three animals, and inability to move in three animals, resulting from hind limb and fore limb impairment in these rats. Treatment with C_60_ at the both tested treatment regimen showed improvement in behavioral changes when compared with 3-NPA-alone-treated animals. None of the rats in C_60_-treated groups showed hind limb paralysis and inability to move, which indicates C_60_ potent activity in reversing 3-NPA- induced motor abnormalities.

### 2.3. Oxidative Status of the Brain Mitochondria after Acute 3-NPA Exposure and C_60_ Supplementation

**Figure 2:**
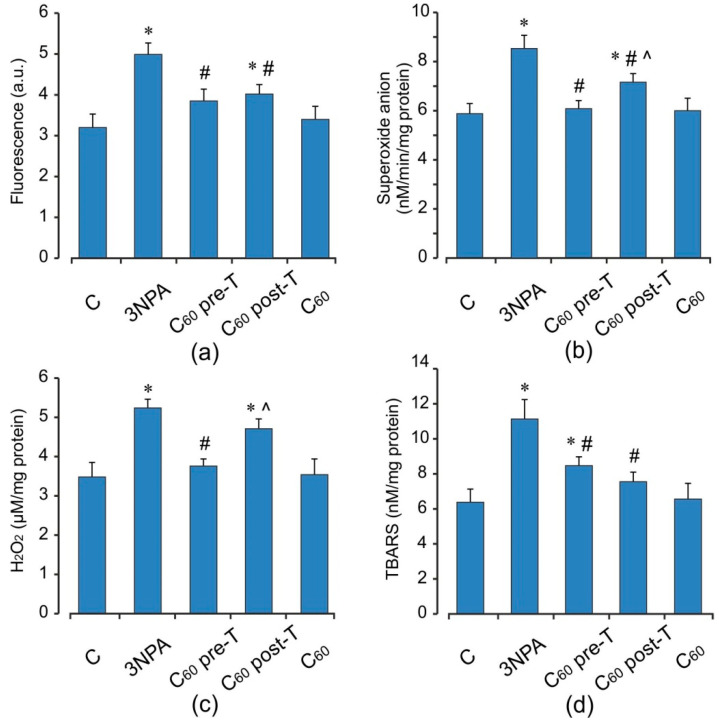
Effect of C_60_ administration on oxidative stress markers: ROS formation (**a**), O_2_^•−^ (**b**), H_2_O_2_ (**c**), and TBARS (**d**) production in brain mitochondria of 3-NPA-treated rats. Values are means ± SD (replicates = 8). The data were analyzed for statistical significance using ANOVA followed by the Bonferroni post hoc test. (C)—control; (3NPA)—3-nitropropionic acid treated rats; (C_60_ pre-T)—C_60_ administration in pre-treatment regimen; (C_60_ post-T)—C_60_ administration in post-treatment regimen; (C_60_)—C_60_ administration alone. * *p* < 0.05 vs. control group; ^#^ *p* < 0.05 vs. 3-NPA treated rats; ^^^ *p* < 0.05 vs. C_60_ pre-T group.

**Figure 3:**
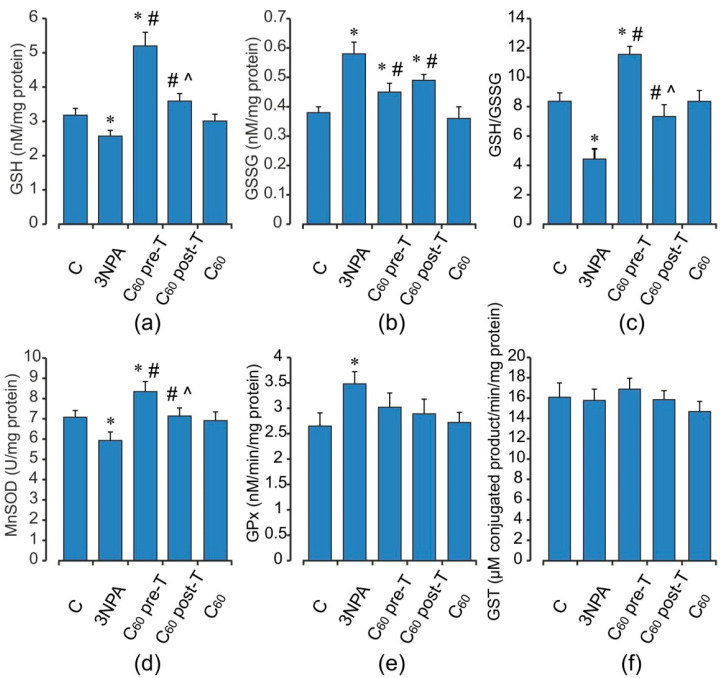
Effect of 3-NPA and C_60_ administration on glutathione pool (**a**–**c**) MnSOD (**d**), GPx (**e**), and GST (**f**) activities in brain mitochondria. Values are means ± SD (replicates = 8). The data were analyzed for statistical significance using ANOVA followed by the Bonferroni post hoc test. (C)—control; (3NPA)—3-nitropropionic acid treated rats; (C_60_ pre-T)—C_60_ administration in pre-treatment regimen; (C_60_ post-T)—C_60_ administration in post-treatment regimen; (C_60_)—C_60_ administration alone. * *p* < 0.05 vs. control group; ^#^ *p* < 0.05 vs. 3-NPA treated rats; ^^^ *p* < 0.05 vs. C_60_ pre-T group.

Animals treated with 3-NPA showed a significant increase in DCF oxidation, an index of reactive oxygen species (ROS) formation, O_2_^•−^ and H_2_O_2_ productions by 56, 45, and 51%, respectively (*p* < 0.05), as well as the TBARS accumulation and the enhancement in GSSG content (by 75 and 53%) (*p* < 0.05) in brain mitochondria when compared with the control group (Figure 2 and Figure 3a–c). These changes were accompanied by a decrease in both MnSOD activity and reduced glutathione level (by 16 and 19%, respectively, *p* < 0.05) (Figure 3). At the same time, the activity of GPx was elevated by 32% (*p* < 0.05), and activity of GST tended to increase than those in the control rats. 3-NPA-induced damage was denoted by reduced GSH/GSSG ratio in 1.9-fold as compared to control (*p* < 0.05). Pre- and post-treatment with C_60_ significantly reduced the production of ROS (by 23 and 19%), O_2_^•−^ (by 29 and 16%), and TBARS (by 24 and 32%) in brain mitochondria, respectively (*p* < 0.05). Pre-treatment with C_60_ significantly decreased the H_2_O_2_ concentration by 28% (*p* < 0.05), whereas the index of H_2_O_2_ generation after C_60_ post-treatment was unchanged in comparison with the 3-NPA group alone and was higher than the control by 35% (*p* < 0.05) (Figure 2). Applications of C_60_ (in both pre- and post-treatment groups) elevated GSH content 2 and 1.4-fold, GSH/GSSG ratio by 22 and 15%, as well as activity of MnSOD by 41 and 21%, respectively, (*p* < 0.05), in comparison with the 3-NPA rats, and maintained GPx and GST activities on the optimal control level (Figure 3). Interestingly, C_60_ pre-treatment supplementation was more successful in preventing oxidative stress in brain mitochondria than C_60_ post-treatment administration, due to a decrease observed in O_2_^•−^ and H_2_O_2_ generation as well as an enhancement in GSH content, GSH/GSSG ratio, MnSOD, and SDH activities to a greater extent than in C_60_ post-treatment (*p* < 0.05).

### 2.4. Oxidative Status of the Skeletal Muscle Mitochondria after Acute 3-NPA Exposure and C_60_ Supplementation

**Figure 4:**
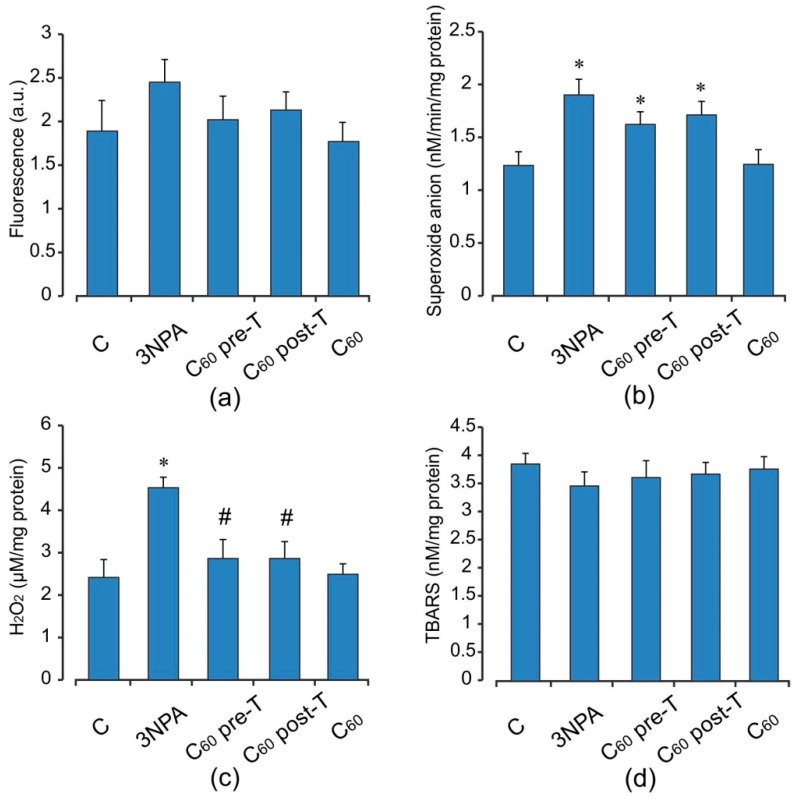
Effect of C_60_ administration on oxidative stress markers: ROS formation (**a**), O_2_^•−^ (**b**), H_2_O_2_ (**c**), and TBARS (**d**) production in skeletal muscle mitochondria of 3-NPA-treated rats. Values are means ± SD (replicates=8). The data were analyzed for statistical significance using ANOVA followed by the Bonferroni post hoc test. (C)—control; (3NPA)—3-nitropropionic acid treated rats; (C_60_ pre-T)—C_60_ administration in pre-treatment regimen; (C_60_ post-T)—C_60_ administration in post-treatment regimen; (C_60_)—C_60_ administration alone. * *p* < 0.05 vs. control group; ^#^ *p* < 0.05 vs. 3-NPA treated rats.

**Figure 5:**
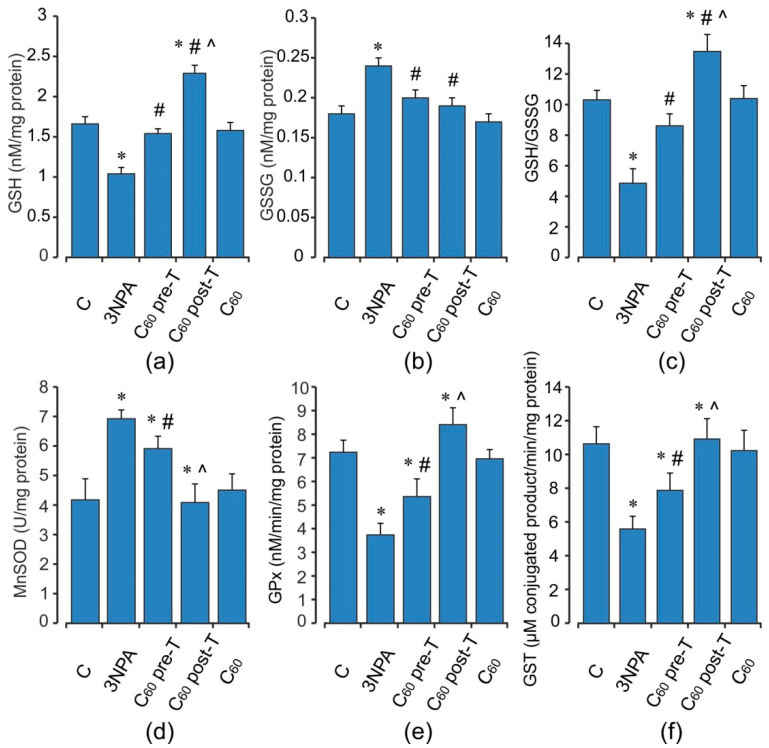
Effect of 3-NPA and C_60_ administration on glutathione pool (**a**–**c**) and MnSOD (**d**), GPx (**e**), and GST (**f**) activities in skeletal muscle mitochondria. Values are means ± SD (replicates = 8). The data were analyzed for statistical significance using ANOVA followed by the Bonferroni post hoc test. (C)—control; (3NPA)—3-nitropropionic acid treated rats; (C_60_ pre-T)—C_60_ administration in pre-treatment regimen; (C_60_ post-T)—C_60_ administration in post-treatment regimen; (C_60_)—C_60_ administration alone. * *p* < 0.05 vs. control group; ^#^ *p* < 0.05 vs. 3-NPA treated rats; ^^^ *p* < 0.05 vs. C_60_ pre-T group.

Acute 3-NPA intoxication stimulated in muscle mitochondria ROS, O_2_^•−^ and H_2_O_2_ production (by 30, 54, and 88%, respectively) without changes in TBARS content comparing to the control rats (*p* < 0.05) (Figure 4). In turn, in muscle mitochondria, in response to these alterations, there was a significant decrease in the activity and content of endogenous antioxidants, including GPx (by 48%), GST (by 47%), and GSH (by 37%) with concomitant increase in MnSOD activity (by 66%) and GSSG level (by 34%) compared to the control groups (*p* < 0.05). The GSH/GSSG ratio was diminished 2.1-fold in comparison with the control (Figure 5). Supplementation of C_60_ in both pre- and post-treatment groups induced a decrease the free radical level (ROS and O_2_^•−^ generation) and the intensity of lipid peroxidation in muscle mitochondria, but these differences were not statistically significant in comparison with the 3-NPA-alone group. Furthermore, H_2_O_2_ concentration in the indicated groups above was reduced by 37 and 42% (*p* < 0.05) compared to the 3-NPA-alone group (Figure 4). In contrast to brain mitochondria, we observed significant induction in GSH content and activity of GSH-related enzymes. Thus, C_60_ administration in pre- and post-treatment groups significantly increased GSH level (in 1.43 and 2.2-fold), GSH/GSSG ratio (in 1.8 and 2.8-fold), as well as activity of GPx (in 1.9 and 2.6-fold) and GST (in 1.4 and 1.96-fold), respectively, and decreased GSSG content (by 16 and 21%) when compared to the 3-NPA-alone group (*p* < 0.05). In addition, C_60_ application (in both treatment regimens) diminished the 3-NPA-induced over-activation of MnSOD (Figure 5). It should be noted that in muscle mitochondria, C_60_ supplementation in post-treatment regimen showed more strengthening of the capacity of GSH pool and GSH-related enzymes than in the pre-treatment regimen.

### 2.5. Activity of Electron Transport Chain Enzymes in Brain and Skeletal Muscle Mitochondria after Acute 3-NPA Exposure and C_60_ Supplementation

**Figure 6:**
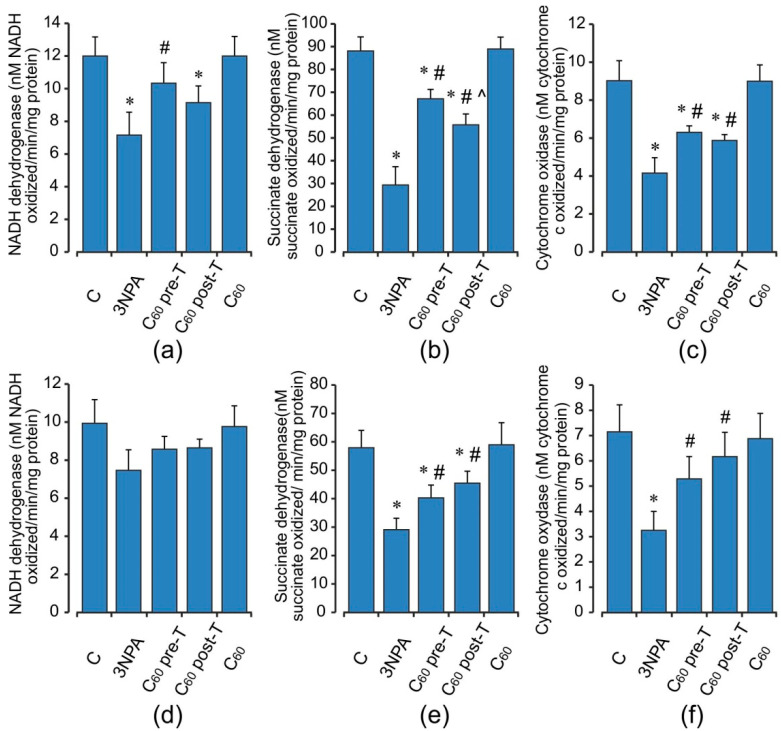
Effect of C_60_ and 3-NPA administration on the activity of the respiratory chain enzymes: NADH dehydrogenase (**a**,**d**), Succinate dehydrogenase (**b**,**e**), Cytochrome oxidase (**c**,**e**) in brain (**a**–**c**) and skeletal muscle (**d**–**f**) mitochondria. Values are means ± SD (replicates=8). The data were analyzed for statistical significance using ANOVA followed by the Bonferroni post hoc test. (C)—control; (3NPA)—3-nitropropionic acid treated rats; (C_60_ pre-T)—C_60_ administration in pre-treatment regimen; (C_60_ post-T)—C_60_ administration in post-treatment regimen; (C_60_)—C_60_ administration alone. * *p* < 0.05 vs. control group; ^#^ *p* < 0.05 vs. 3-NPA treated rats; ^^^ *p* < 0.05 vs. C_60_ pre-T group.

The activities of mitochondrial electron chain enzymes in the brain and muscle are presented in Figure 6. Brain mitochondrial dysfunction induced by 3-NPA administration at high doses exhibited a significant decrease in activities of mitochondrial enzyme complexes (I, II, and IV) as compared to the vehicle/control group. Thus, the activities of NADH dehydrogenase, SDH, and cytochrome oxidase were decreased by 40, 66, and 54% (*p* < 0.05). The activity of SDH was reduced to a greater extent as compared to other complexes and this might be because of the irreversible inhibition of complex II by 3-NPA [7,10]. Pre- and post-treatment with C_60_ counteracted the deleterious effect of 3-NPA by increasing the mitochondrial enzyme complexes’ activities in comparison with the 3-NPA-alone group. In skeletal muscle, 3-NPA treatment caused significant inhibition activities of SDH and cytochrome oxidase, but the activities of NADH dehydrogenase were not altered significantly. C_60_ treatment restored the activity of complexes I, II, and IV as compared to 3-NPA injection. These effects were similar in pre- and post-treatment groups.

### 2.6. Effect of 3-NPA Exposure and C_60_ Supplementation on Pro- and Anti-Apoptotic Markers

**Figure 7:**
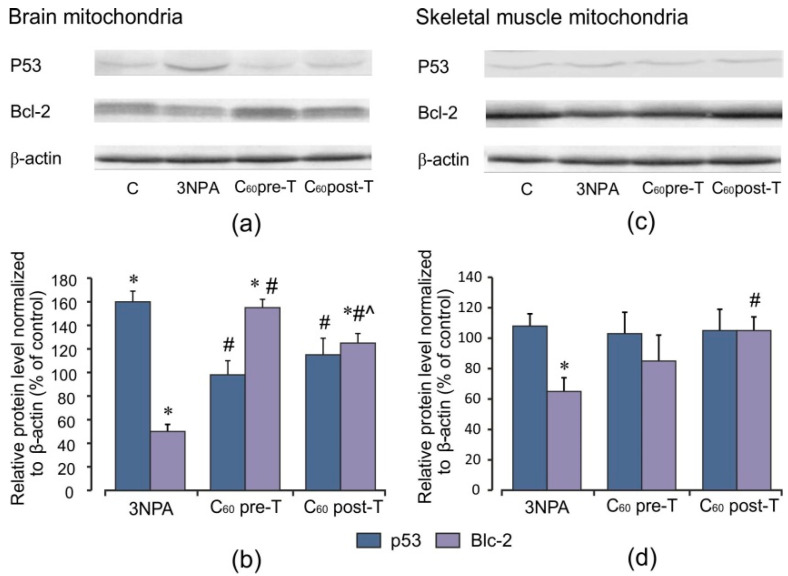
Effect of C_60_ and 3-NPA administration on p53 and Bcl-2 protein expression in brain and skeletal muscle mitochondria. (**a**,**c**) Representative Western blot and (**b**,**d**) densitometric analysis of p53 and Bcl-2 protein contents. Protein extracts were separated by performing SDS PAGE and subsequently electroblotted onto PVDF membranes. The values of the p53 and Bcl-2 proteins were normalized to β-actin. Final Western blot figure as the histogram is expressed as mean percentages (±SD) over control values from two independent experiments. The control values are taken as 100%. Statistically significant differences are indicated as * *p* < 0 05 vs. control; ^#^ *p* < 0 05 vs. 3-NPA treated rats; ^^^ *p* < 0.05 vs. C_60_ pre-T group.

Western blotting analyses for p53 as a transcription factor regulating apoptosis and Bcl-2 as an anti-apoptotic agent were performed to better understand the mechanisms mediating the 3-NPA-induced oxidative stress disorders and the protective effects afforded by C_60_. As shown in Figure 7, 3-NPA treatment triggered an increase in the protein content of p53 in brain mitochondria by 60% (*p* < 0.05) and a decrease in Bcl-2 protein level by 50% (*p* < 0.05) in comparison with the control. In skeletal muscle, we registered only minor indication in p53 mitochondrial accumulation; at the same time, Bcl-2 protein expression was diminished by 35% (*p* < 0.05). C_60_ application induced a decrease in p53 protein content in brain and muscle mitochondria compared to the control level in both treatment regimens. C_60_ injection in pre- and post-treatment regimens caused elevation of the Bcl-2 protein level in brain mitochondria by 55 and 25% (*p* < 0.05) and in skeletal muscle mitochondria by 15% (*p* > 0.05) and 40% (*p* < 0.05), respectively, compared to the 3-NPA-alone group, showing C_60_ anti-apoptotic effect. In the brain, we found a positive correlation between p53 protein content and ROS formation (*r* = 0.67; *p* < 0.05) and negative correlations between p53 protein content and MnSOD activity as well as MnSOD protein level (*r* = −0.75 and *r* = −0.58, respectively; *p* < 0.05).

### 2.7. Effect of 3-NPA Exposure and C_60_ Supplementation on Protein Expression of Nrf2 and Nrf2 Target Proteins

**Figure 8:**
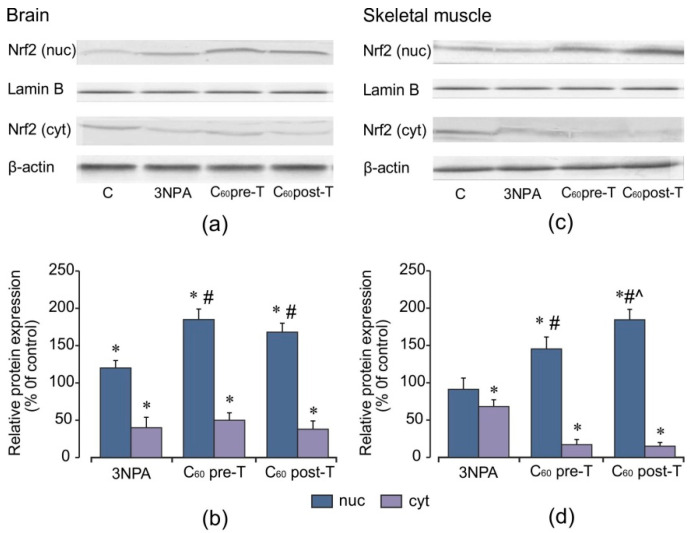
Effect of C_60_ and 3-NPA administration on the nuclear and cytosolic Nrf2 protein expressions in the brain and skeletal muscle cells. (**a**,**c**) Representative Western blot and (**b**,**d**) densitometric analysis of the Nrf2 protein contents. Protein extracts were separated by performing SDS PAGE and subsequently electroblotted onto PVDF membranes. The values of the nuclear and cytosolic Nrf2 proteins were normalized to Lamin B and β-actin, respectively. Final Western blot figure as the histogram is expressed as mean percentages (±SD) over control values from two independent experiments. The control values are taken as 100%. Statistically significant differences are indicated as * *p* < 0 05 vs. control; ^#^ *p* < 0 05 vs. 3-NPA treated rats; ^^^ *p* < 0.05 vs. C_60_ pre-T group.

**Figure 9:**
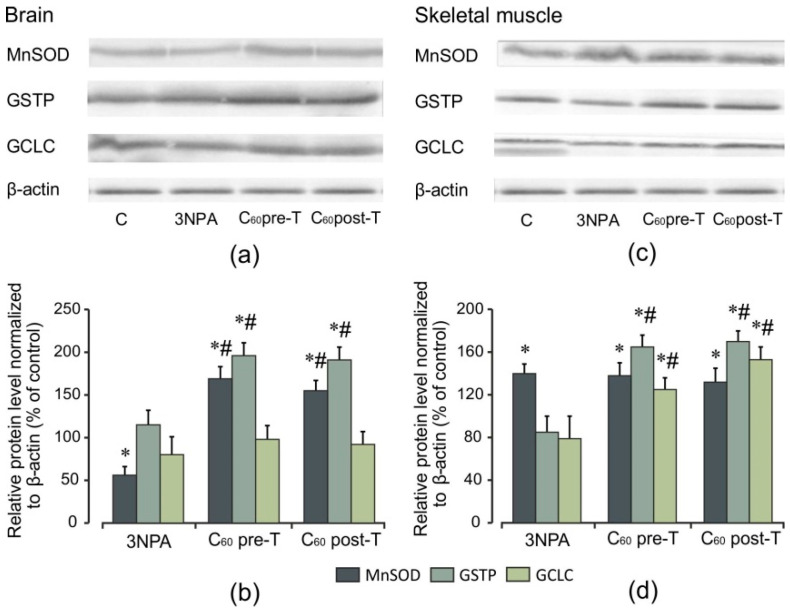
Effect of C_60_ and 3-NPA administration on MnSOD, GSTP, GCLC protein expressions in brain and skeletal muscle mitochondria. (**a**,**c**) Representative Western blot and (**b**,**d**) densitometric analysis of protein levels. Protein extracts were separated by performing SDS PAGE and subsequently electroblotted onto PVDF membranes. The values of the proteins were normalized to β-actin. Final Western blot figure as the histogram is expressed as mean percentages (±SD) over control values from two independent experiments. The control values are taken as 100%. Statistically significant differences are indicated as * *p* < 0 05 vs. control; ^#^ *p* < 0 05 vs. 3-NPA treated rats.

The fact that ROS leads to neurodegeneration [2] and antioxidant therapy has neuroprotective effects in HD [3] may point to a potential beneficial effect of the Nrf2 pathway. To investigate whether C_60_ treatment modulate keys proteins involved in the cellular response to oxidative stress, the levels of Nrf2 protein expression and its downstream targets were evaluated by Western blot analysis. 3-NPA exposure at a high dose results in an increase (by 20%) in Nrf2 nuclear protein levels in the brain, with a concomitant decrease in cytosolic Nrf2 protein levels (*p* < 0.05). In contrast, in the skeletal muscle, no significant changes in these indices were observed in comparison to the control (Figure 8). As shown in Figure 9, in the brain, 3-NPA triggered a decrease in the protein content of MnSOD by 44% (*p* < 0.05), but in skeletal muscle, the MnSOD protein level was significantly enhanced (by 40%, *p* < 0.05) in comparison to the control group. The protein contents of GCLC and GSTP were close to the control level *(p* > 0.05) and these changes were similar in both tissues. We found that C_60_ administration in both tested regimens caused a significant elevation in the Nrf2 protein level in both the brain and skeletal muscle nuclear extracts in comparison to the control and 3-NPA-alone groups (*p* < 0.05), which was accompanied by a decrease in the Nrf2 cytosolic protein expression (*p* < 0.05) indicating translocation of Nrf2 from cytosol to the nucleus. Together with the increase in nuclear Nrf2 levels, we registered a statistically significant increase in the protein expression of MnSOD and GSTP in the brain as well as of GSTP and GCLC in the skeletal muscle cytosolic fractions relative to the 3-NPA-alone and control groups (*p* < 0.05). In the brain, the GCLC protein content is kept at the control level. The above indices are expressed similarly in both tested C_60_ treatment regimens. There were positive correlations between protein content of Nrf2 and GSTP (*r* = 0.80; *p* < 0.05) and protein content of Nrf2 and MnSOD (*r* = 0.61; *p* < 0.05) in the pre-T group as well as in the post-T group (*r* = 0.49; *p* < 0.05) in the brain. For skeletal muscle, positive correlations were observed between protein content of Nrf2 and GSTP (*r* = 0.61; *p* < 0.05) and protein content of Nrf2 and GCLC (*r* = 0.50; *p* < 0.05) in the C_60_ pre-T regimen. In the C_60_ post-T group, positive correlations were found between protein content of Nrf2 and GSTP (*r* = 0.85; *p* < 0.05) and protein content of Nrf2 and GCLC (*r* = 0.79; *p* < 0.05). These findings correlate with the rise in GSH content (Figure 3a–c and Figure 6a–c). Thus, positive correlations were found between the protein content of Nrf2 and GSH level (*r* = 0.88; *p* < 0.05) in the brains of rats after C_60_ administration in the pre-T regimen and between the protein content of Nrf2 and GSH level (*r* = 0.91; *p* < 0.05) in the skeletal muscle of rats in the C_60_ post-T regimen. Our results suggest that the Nrf2 pathway takes part in GSH synthesis in the brain and skeletal muscle tissues under C_60_ supplementation. 

### 2.8. Effect of C_60_ Supplementation Alone on Oxidative Stress Markers and Indices of Antioxidant Defense

C_60_ treatment alone did not produce any significant effect on the oxidative stress markers, antioxidant status, and mitochondrial enzyme complexes activity in the brain and skeletal muscle mitochondria as compared to the control group (Figure 2, Figure 3, Figure 4, Figure 5, Figure 6). In our early study, we demonstrated that in investigated tissues, the protein level of Nrf2 in the nuclear and cytosol fractions was close to the control level. The protein content of MnSOD and GSH-related enzymes in tissues tended to increase, but this effect had no statistical significance [23]. The data indicated that the investigation of the protein level of Nrf2 and its target proteins after C_60_ supplementation alone was not necessary for our present study.

## 3. Discussion

In the present study, the administration of 3-NPA at high doses triggered the series of events, including the decreases in activity of complexes-I, -II, and -IV, increases in ROS, O_2_^•−^, and H_2_O_2_ formation, and the consequent rise in TBARS production as well as disturbance of antioxidant homeostasis, which ultimately led brain mitochondria to a dysfunctional state. The intensification of oxidative processes in brain mitochondria was accompanied by the increase of GSSG content and decrease of GSH concentration as well as decrease of GSH/GSSG ratio, which were additional important indicators of oxidative stress and mitochondrial dysfunction [13,15]. Our findings were in concordance with previous studies where the disturbances in mitochondrial bioenergetics, defects in mitochondrial complexes II–IV, F_1_F_0_ ATP-ase, and aconitase, mitochondrial swelling, disbalance in the pro-antioxidant system, excessive mitochondrial fission, and subsequent neuronal cell death in different regions of the brain after prolonged and acute 3-NPA treatment have been demonstrated [4,38,39,40]. Numerous pieces of evidence from clinical and experimental studies validated the key role of oxidative stress and concomitant mitochondrial dysfunction in mediating the neuronal degeneration and behavioral abnormalities at HD as well as after the exposure to neurotoxin 3-NPA [3,6,8,38,39].

Among the enzymes of antioxidant defense that play important roles in the removal of ROS excess in living organisms, SOD and GPx are the most important. These enzymes work in an interrelationship to eliminate reactive oxygen species, and even slight alterations in their action cause a dramatic effect on the resistance of the cells to oxidative damage [14]. GSH as a non-enzymatic antioxidant is especially crucial in mitochondria protection from xenobiotic- and ROS-induced toxicity [13,15]. In brain mitochondria, we registered decreased activity of MnSOD; it could be increased superoxide radical and peroxynitrite formation that induced MnSOD inactivation [40]. Glutathione depletion registered in our study seemed to be caused by reactions of conjugation with the involvement of the GST or by GSSG formation through the increased H_2_O_2_ production and rise of GPx activity. Our results were in satisfactory concordance with previous reports that acute exposure to 3-NPA was linked to the depletion of thiols in the rat brain together with decreased activities of mitochondrial SOD and catalase [39,41,42]. In addition, proteomic analysis of postmortem human HD striatum and cortex demonstrated increased expression of the antioxidant enzymes peroxiredoxins 1, 2, and 6 and glutathione peroxidases 1 and 6 relative to control samples [43], suggesting ongoing responses to oxidative stress in HD. 

In contrast, in skeletal muscle, the acute 3-NPA intoxication in parallel with the rise of ROS, O_2_^•−^, and H_2_O_2_ generation progressively increased the MnSOD activity and protein expression in mitochondria. These effects could be explained by a compensatory increase in this enzyme activity in response to the increased superoxide anion production, which serves as an MnSOD substrate [1,14]. The induction of MnSOD under such conditions could be seen as a defensive reaction to the excess production of active oxygen metabolites. The last ones, in turn, could activate the expression of antioxidant enzymes via different signaling pathways [44,45]. Indeed, MnSOD is known as an inducible enzyme that may be activated in various stressful conditions [45,46]. It is well known that over-expression of MnSOD can cause protection against oxidative stress-induced disorders [44,45]. In this context, the induction of ROS-scavenging enzyme can attenuate the lipid peroxidation that we observed in skeletal muscle mitochondria, saving them from potential cell damage and death.

Nevertheless, overexpression of MnSOD without the appropriate increase of GPx and GST levels, which we demonstrated in our study, resulted in the H_2_O_2_ accumulation that disrupted the mitochondrial glutathione redox status [14,15,44]. The decline in the content of GSH, GPx, and GST activities that was registered in skeletal muscle mitochondria of 3-NPA-treated rats might be linked with the increased utilization of antiperoxide enzymes to counteract the high level of lipid peroxidation and oxidative injury induced by the toxin.

In our study, for correction of mitochondrial dysfunction in rats exposed to 3-NPA two schedules of C_60_ introduction were used. We had shown that pre- and post-treatment with C_60_ induced the reduction of lipid peroxidation intensity. It could be explained by the inhibition of ROS production, namely, superoxide anion release and H_2_O_2_ generation, which we registered in mitochondria. 

We can suppose that in our study, C_60_ realized its antioxidative activity via several mechanisms, such as direct scavenging of free radicals and restoration of mitochondrial enzyme complexes’ activities, especially SDH, which was the main target for neurotoxin 3-NPA [7,8,10]. These mechanisms may contribute to C_60_ potent antioxidative and assumed neuroprotective activities. We had registered that C_60_ normalized the altered antioxidant defense system in mitochondria of both studied tissues by modulation of the enzymatic and non-enzymatic antioxidant levels. Thus, in brain mitochondria, the coordinated upregulation of MnSOD activity and GSH content, as well as the maintenance of GPx and GST activity on the control level by C_60_ treatment formed a logical pathway for protection against 3-NPA-induced mitochondrial dysfunction. It should be noted that C_60_ application showed a more beneficial effect in the pre-T regimen in brain mitochondria. In contrast, in skeletal muscle mitochondria, the MnSOD hyperactivity loss and GPx and GST enzymes’ capacity strengthening demonstrated C_60_ protective effects manifestation in a post-T regimen.

Neurodegenerative disorders are characterized by progressive dysfunction and loss of neurons in specific and selective areas of the brain [2,3,47]. In the present study, 3-NPA application in a high dose caused body weight decrease and movement abnormalities in rats, which were improved significantly by C_60_ treatment in both pre- and post-treatment regimens. A resemblance to the beneficial effect of C_60_ on skeletal muscle functions and structure was noted in our recent studies where we have shown a significant decrease in skeletal muscle fatigue during prolonged and intense physical loads [24,25] and an increase in the movement dynamics of the hemiparkinsonian rats after C_60_ injection [48].

Mitochondrial dysfunction resulted in the release of factors that initiated and amplified numerous signals that led to apoptosis [8]. P53 as a transcription factor plays a central role in the regulation of apoptosis through transcription-dependent and -independent mechanisms [36]. Recent studies demonstrated a novel function of p53 in retaining redox homeostasis through the regulation of energy metabolism, mitochondrial biogenesis, and expression of antioxidant enzymes [37,49].

In our study, the rats exposed to a high dose of 3-NPA at the same time as oxidative stress intensification demonstrated alterations in the p53 protein content in both cell compartments (cytosol and mitochondria), and these changes were tissue-specific. The disappearance of p53 protein from a cytoplasm after 3-NPA exposure resulted in p53 protein accumulation in mitochondria and perhaps in the nucleus, because it seems that p53 translocation to mitochondria precedes its nuclear localization [49]. Thus, in brain mitochondria, the p53 protein content was significantly elevated, while in muscle mitochondria, this index had the only tendency to enhancement. It should be noted that in the brain, the level of ROS formation positively correlated with the mitochondrial p53 protein content, indicating that p53 cell distribution may be ROS-regulated. Our findings are in agreement with previous reports where 3-NPA induced an accumulation of p53 in the brain cells and ROS level was the main modulator of p53 stability and functions under stressful conditions [9,35,41,50].

Recent studies indicated that p53 protein translocation to mitochondria can induce transcription-independent apoptosis through the direct interaction with the Bcl-2 proteins family, which are located in the outer mitochondria membrane [51]. Anti-apoptotic Bcl-2 proteins serve as a known sensor of apoptotic signaling by blocking pro-apoptotic cytochrome c release from mitochondria, and in that way, prevent apoptosis [51,52].

In parallel with p53 mitochondrial translocation, we had registered the decrease in Bcl-2 protein levels in brain and muscle mitochondria. These findings demonstrated that 3-NPA can induce apoptotic cascade activation in mitochondria through the increase in p53 protein level and decrease in Bcl-2 protein content, and these changes had different degrees of manifestation depending on tissue type. Indeed, in 3-NPA models, upregulation of pro-apoptotic Bax and Bak and downregulation of Bcl-2 and Bcl-xl resulted in a marked disruption of mitochondrial membranes with subsequent cytochrome c release and procaspase-3 activation [4,41,53].

In our study, mitochondrial p53 protein content negatively correlated with MnSOD activity. We can suppose that after 3-NPA application, p53 protein level actively influenced MnSOD-specific activity/protein content in brain mitochondria. Our findings were in agreement with studies that describe MnSOD as a downstream target of p53, which can be specifically downregulated [49,50,54]. As it was previously reported by other investigators, p53 not only inhibits MnSOD superoxide scavenging activity by physically interacting with the enzyme in mitochondria. It is also linked to the regulation of MnSOD protein levels since p53 played a dual role: At a low concentration, p53 increased MnSOD protein level, whereas at a high concentration, p53 decreased MnSOD protein expression [54]. However, the actual mechanism of MnSOD regulation could be more complicated with the involvement of other possible “players”. Thus, St-Pierre et al. showed that the decrease in transcription and activity of peroxisome proliferator-activated receptor gamma coactivator 1-alpha (PGC-1 α) in an HD mice model and the brain tissues from HD patients led to the downregulation of genes encoding SOD1, SOD2, and glutathione peroxidase, resulting an increased oxidative damage and neuronal death [55].

Because of their antioxidant properties, fullerenes are involved in the inhibition of apoptosis in various cell types including neuronal cells [22,30]. In our study, C_60_ injection in both treatment regimens decreased the mitochondria-dependent apoptosis in the mitochondria of rats exposed to 3-NPA by limiting p53 translocation as well as by enhancing Bcl-2 protein expression. This possibility is supported by recent studies indicating that Bcl-2 overexpression limits apoptosis by preventing ROS-induced mitochondrial permeability transition pore opening through the direct interaction with pro-apoptotic Bax/Bak or BH3-only proteins [9,56]. Besides its direct antiapoptotic role, Bcl-2 participated in maintaining redox homeostasis by regulating glutathione and NADPH levels. Bcl-2 overexpression also results in a shift in the cellular redox state toward a more reduced level [56]. The latter is important because the cellular thiol redox state can regulate programmed cell death [52]. A decreased intracellular GSH/GSSG ratio causes anti-apoptotic protein Bcl-2 loss, cytochrome c release from mitochondria, and caspase activation by the induction of the p38 mitogen-activated protein kinase pathway, whereas an increased intracellular GSH/GSSG ratio prevents the programmed cell death [51,52,56]. We supposed that an increase in mitochondrial glutathione production, GSH/GSSG ratio, as well as Bcl-2 protein content by C_60_ could be among the protective mechanisms by which nano-carbon had limited apoptotic effects in mitochondria. 

We previously reported that C_60_ effects on oxidative stress protection in 3NPA-induced neurodegeneration through its antioxidant properties. However, the mechanisms of C_60_ are still poorly described. In the present study, we investigated the potential molecular mechanisms underlying the C_60_ protective effect in 3-NPA-induced neurodegeneration through the activation of the Nrf2 /ARE pathway.

During recent years, some studies have highlighted the protective effects of Nrf2 activation in reducing oxidative stress in both in vitro and in vivo models of neurodegenerative disorders [57,58,59]. Calkins et al. demonstrated that Nrf2 is an essential inducible factor for protection against complex II inhibitor-mediated neurotoxicity; and Nrf2-mediated ARE transcription is a potential strategy for preventive therapy in neurodegenerative diseases, like HD [58]. According to several in vitro models, Nrf2 activation eliminated mHtt-induced toxicity since co-transfection of Nrf2 with mHtt can decrease the mean lifetime of mHtt N-terminal fragments and improved cell viability in primary striatal neurons [59].

Recently, it has been demonstrated that the Nrf2 activates different genes that encode various antioxidants and phase 2 detoxification enzymes [17] and the GSH redox cycle could be one of its targets. 

In our study, a high dose of 3-NPA caused an increase of Nrf2 nuclear protein content in the brain tissue with a concomitant decrease in the cytosol fraction. However, in skeletal muscle tissue, no significant changes in Nrf2 nuclear protein level were observed. The protein expression of Nrf2 targets GSTP and GCLC were close to the control level in both tissues of rats that were exposed to 3-NPA.

Nrf2 is sequestered in the cytoplasm as an inactive complex with its cytosolic repressor Kelch-like ECH-associated protein-1(Keap-1). It is known that in presence of electrophiles or oxidative stress, the nucleophilic cysteine sulfhydryl groups in Keap-1 are modified, resulting in allosteric conformational alteration that diminishes KEAP-dependent degradation of Nrf2 and allows the transcription factor to be accumulated in the nucleus [60]. Moreover, along with Nrf2 activation mode, there are other mechanisms, including phosphorylation by different kinases, which can also result in its dissociation from Keap-1 and increased nuclear localization [16,17,57,60]. We consider that in our case, Nrf2 activation with an accumulation of Nrf2 protein in the nucleus, can result from 3-NPA-induced ROS overproduction, especially O_2_^•−^, GSH/GSSG redox alteration, loss of ATP, or even as a direct response to SDH inhibition. Our findings coincide with previous reports that acute and prolonged 3-NPA administration provokes Nrf2 activation in neuronal cells [38,53,61] and this process was ROS-regulated [16,57].

The present study demonstrated that C_60_ administration evoked further enhancement of nuclear Nrf2 protein in the brain and skeletal muscle of rats incurred by 3-NPA with an associated decrease in the cytosol fraction. The levels of protein expression of GSH-related enzymes correlated positively with Nrf2 nuclear protein level, supposing that the upregulation of GCLC and GSTP can depend on the Nrf2/ARE pathway. We have assumed that C_60_ influenced GSH recycle via the induction of γ-GCLC and GSTP protein expressions and this is necessary for the recovery of GSH system homeostasis. 

Therefore, C_60_ treatment led to significantly increased levels/activities of MnSOD, GSH, and GST in the mitochondrial fraction. We supposed that the increased synthesis of GSH as well as MnSOD and GSH-related enzymes in the cytoplasm could simultaneously increase the level of these antioxidants in the mitochondrial compartment. Since mitochondria are crucial intracellular targets for oxidant cell damage [11], our findings are important for understanding the phenomena of C_60_ action leading to cyto- and mitoprotection against 3-NPA-induced oxidative stress in the brain and muscle cells.

## 4. Materials and Methods

### 4.1. Material Preparation and Characterization

A stable colloid solution of fullerene C_60_ in dimethyl sulfide (DMSO) was prepared as previously described in Maznychenko et al. [62]. Briefly, DMSO (purity > 99.99%, Sigma-Aldrich, Germany) was added to the pure fullerene C_60_ powder (Sigma-Aldrich, Germany, purity > 99.99%) at a final concentration of 1 mg/mL. Further, the mixture was treated in an ultrasonic bath for approximately 35–45 min. The procedure was continued until a visible, brown-colored solution was obtained. In order to monitor the quality of the fullerene C_60_-DMSO solution, UV-vis spectrophotometry, followed by atomic force microscopy (AFM) was used. Registered absorbance spectra, as well as measured sizes of the dispersed fullerene C_60_ nanoparticles, were consistent with the previously reported data [62].

### 4.2. Experimental Design and Sample Collection

Fifty male Wistar rats weighing 220–260 g were used in the study. The body weight of animals was recorded on the first and last day of the experiment. The rats were housed in plexiglas cages (4 rats per cage) and maintained in an air-filtered and temperature-controlled (20–22 °C) room. Rats received a standard pellet diet and water ad libitum. The use of the animals was approved by the Ethics Committee of the Institute and performed under the European Union Directive of 22 September 2010 (2010/63/EU) for the protection of animals used for scientific purposes. All chemicals were purchased from Sigma-Aldrich Inc., St. Louis, MO 63103, USA and were of the highest purity.

Drugs and Treatment Schedule

3-NPA was diluted in buffered saline (pH 7.4) and administered intraperitoneally (i.p.) at a dose of 30 mg/kg once a day for 3 days to induce HD-like symptoms. The 3-NPA dose was chosen based on preliminary studies in which biochemistry alterations characteristic of 3-NPA neurotoxicity were observed [38,42,63].

The animals were treated i.p. with C_60_ fullerene (C_60_) in DMSO solution dissolved in distilled water at a dose of 0.5 mg/kg once a day. The C_60_ dosage was based on previous studies where the safety profile was checked and found to be nonlethal. No toxic effects or deaths have been fixed under the action of C_60_ after i.p. injection at a dose of 0.5 mg/kg [23,25]. Rats were divided into five groups (*n* = 10/group) based on treatment regimen (see also Table 2): Group 1 received vehicle and served as a control (C); group 2 received 3-NPA alone for 3 consecutive days (3-NPA); group 3 (C_60_ fullerene pre-treatment) received C_60_ (0.5 mg/kg) 2 days prior to the beginning of 3-NPA treatment and 3 days along 3-NPA treatment, for 5 consecutive days in total (C_60_ pre-T); group 4 (C_60_ fullerene post-treatment) received C_60_ (0.5 mg/kg) after 3-NPA treatment for 5 consecutive days (C_60_ post-T); group 5 received only C_60_ (0.5 mg/kg) for 5 consecutive days.

The controls/vehicles were administrated i.p. with an equal volume of DMSO solution dissolved in distilled water once a day along the same period. Note that in all cases, the final DMSO concentration in the injected solutions was not more than 1%.

Drugs were administered i.p. between 9:00 a.m. and 10:00 a.m. once a day. C_60_ was given 1 h before the 3-NPA exposure.

### 4.3. Neurological Scoring (Movement Analysis)

To assess the severity of 3-NPA-induced neurological disorders, animals were evaluated on the first and last day of the experiment according to the following characteristics: 0—normal behavior, 1—general slowness of displacement resulting from mild hind limb impairment, 2—incoordination and marked gait abnormalities, 3—hind limb paralysis, 4—incapacity to move resulting from fore limb and hind limb impairment as described by Ludolph [10].

### 4.4. Sample Collection and Mitochondria Isolation

Animals were decapitated 24 h after the last drug injection. At the time of sacrifice, the animals were lightly anesthetized with ether. All solutions, glassware, centrifuge tubes, and equipment were pre-cooled to 0 °C to 4 °C and kept on ice.

#### 4.4.1. Isolation of Rat Brain Mitochondria

The whole brain was rapidly removed and freed from blood and vessels. Rat brain mitochondria were isolated by differential centrifugation according to the method of Sims [64]. The tissue was finely minced with scissors in a small amount of isolation buffer (0.32 M sucrose, 20 mM HEPES, 1 mM EGTA, 10 mM Tris/ HCI, pH 7.4) and washed three times with this buffer. The tissue in isolation buffer (l0%, *wt/vol*) was homogenized. The homogenate was centrifuged at 1330× *g* for 3 min and the supernatant was carefully decanted and the pellet resuspended in half of the original volume. Subsequently, homogenate was re-centrifuged as above, the supernatant was retained, and the pellet discarded. The pooled supernatant was centrifuged at 20,200× *g* for 10 min. The decanted supernatant was discarded, and the pellet resuspended in 15% Percoll. Tubes were centrifuged for 5 min at 21,700× *g*. Three major bands of material were obtained, and the material banding near the interface of the lower two Percoll layers was diluted 1:4 by gently mixing with isolation buffer and then centrifuged at 16,700× *g* for 10 min. The supernatant was removed and the material gently resuspended. Fatty acid-free bovine serum albumin (10 mg/mL) was added, and the mixture was diluted with isolation buffer (final volume 3 mL). After centrifugation at 6900× *g* for 10 min, the supernatant was rapidly decanted, and the pellet gently re-suspended in an isolation buffer without EGTA. This fraction was stored on ice for further investigations.

#### 4.4.2. Isolation of Rat Skeletal Muscle Mitochondria

The excised hind limb skeletal muscles (soleus and gastrocnemius) were rapidly dissected and freed from fat and tendon. Rat skeletal muscle mitochondria were prepared using the method of Graham [65]. Tissue was collected in isolation medium A (0.1 M sucrose, 46 mM KCl, 10 mM Tris/HCl (pH 7.6), 1 mM EGTA, and 0.5% defatted bovine serum albumin) and homogenized. Forty milligrams of Nagarse (0.2 mg/mL final) was added in isolation medium. After centrifugation of the homogenate at 1000× *g* for 5 min, the supernatant was strained on gauze and recentrifuged at 12,000× *g* for 15 min. The supernatant fraction is decanted into a new tube and re-centrifuged as before, in order to remove residual myofibrils. The resulting pellet was resuspended in ice-cold isolation medium B (250 mM sucrose, 10 mM Tris/HCl (pH 7.6), and 0.1 mM EGTA) and a new series of centrifugation at 12,000× *g* for 15 min was performed. The final washing and resuspension of mitochondria were in medium B without EGTA. The washing medium was discarded and then the surface of the tightly packed pellet was rinsed with 0.15 M KCl in order to remove the remainder of the medium. The mitochondrial pellet is finally suspended in 0.15 M KC1 to contain 6–10 mg of mitochondrial protein per milliliter of suspension.

For biochemical analysis, the mitochondrial preparations were analyzed after solubilization in 0.5% deoxycholate for 60 min at 0–4 °C. The protein concentration was estimated using the Bradford method with bovine serum albumin as a standard.

### 4.5. Evaluation of Oxidative Stress Markers

#### 4.5.1. Measurement of Reactive Oxygen Species (ROS) Formation

ROS formation was determined by dichlorofluorescein (DCF) fluorescence [66]. Fresh isolated mitochondria were loaded for 20 min at 37 °C with a nonfluorescent probe (2′,7′-dichlorodihydrofluorescein diacetate, DCFH-DA). The final concentration of DCFH-DA was 3 mM. DCF formation was measured at the excitation wavelength of 488 nm and emission wavelength of 525 nm for 30 min by using a Hitachi F-2000 fluorescence spectrometer. The rate of DCFH-DA conversion to DCF was linear for at least 60 min, corrected with the autooxidation rate of DCFH-DA without protein. All assays were carried out in duplicate. Fluorescence was expressed as arbitrary fluorescence units.

#### 4.5.2. Measurement of Superoxide Radical Production

Mitochondria superoxide production was measured by superoxide dismutase-sensitive ferricytochrome c reduction assays, as described previously [67]. Briefly, equal portions of mitochondria (0.25 mg of protein) were incubated with 50 μM acetylated ferricytochrome c in the presence or absence of superoxide dismutase (400 U/mL). Catalase (125 U/mL) was added to the reaction to remove any H_2_O_2_ that formed. After incubation at 37 °C for 15 min, the reaction was stopped by adding 1 mM N-ethylmaleimide. The reduction of ferricytochrome c was measured by reading absorbance at 550 nm with a spectrophotometer. The amount of O_2_^•−^ release was calculated by dividing the difference in absorbance of the samples with and without superoxide dismutase by the extinction coefficient for the change from ferricytochrome c to ferrocytochrome c (ε = 24 × 10^3^ M^−1^cm^−1^), and the results were expressed as nmol/min/mg protein.

#### 4.5.3. Measurement of Hydroperoxide

The H_2_O_2_ concentration in the tissue homogenates was measured using the FOX method, which is based on the peroxide-mediated oxidation of Fe^2+^, followed by the reaction of Fe^3+^ with xylenol orange (o-cresolsulphonephthalein 3′,3″-bis [methylimino] diacetic acid, sodium salt). To examine the H_2_O_2_ production, 500 μL of the incubation medium was added to 500 μL of the assay reagent (500 μM ammonium ferrous sulphate, 50 mM H_2_SO_4_, 200 μM xylenol orange, and 200 mM sorbitol). The absorbance of the Fe^3+^-xylenol orange complex (A_560_) was detected after 45 min. Standard curves of H_2_O_2_ were obtained for each independent experiment by adding variable amounts of H_2_O_2_ to 500 μL of basal medium mixed with 500 μL of the assay reagent. The data were normalized and are expressed as μM H_2_O_2_ per mg of protein [68].

#### 4.5.4. Lipid Peroxidation Assay

Lipid peroxidation was measured by the formation of thiobarbituric acidreactive substances (TBARS) according to the method of Buege and Aust [69]. TBARS were isolated by boiling tissue homogenates for 15 min at 100 °C with the thiobarbituric acid reagent (0.5% 2-thiobarbituric acid/10% trichloroacetic acid/0.63 mM hydrochloric acid) and measuring the absorbance at 532 nm. The results are expressed as nM/mg of protein using ε = 1.56 × 10^5^ M^−1^cm^−1^.

### 4.6. Evaluation of Biochemical Parameters

#### 4.6.1. Mitochondrial Respiratory Chain Enzymes

NADH dehydrogenase (complex I) activity was determined spectrophotometrically by the method of King and Howard [70]. The method involves catalytic oxidation of NADH to NAD with subsequent reduction of cytochrome c. The reaction mixture contained 0.2 M glycyl glycine buffer (pH 8.5), 6 mM NADH, and 1 mM cytochrome c. The reaction was initiated by the addition of a requisite amount of solubilized mitochondrial sample and followed by absorbance change at 550 nm for 5 min. Results are expressed as nM cytochrome c oxidized/min/mg protein.

Succinate dehydrogenase (Complex-II) activity was measured by following the decrease in absorbance due to the reduction of 2,6-dichloroindophenol at 600 nm (ε = 19.1 × 10^3^ M^−1^cm^−1^). The reaction mixture consisting of 200 mM potassium phosphate buffer (pH 7.5), 200 mM sodium succinate, 100 mM KCN, and 10 mM 2,6-dichloroindophenol and 25 mM phenazine methosulfate [71]. The results are expressed as nM succinate oxidized/min/mg protein.

Cytochrome oxidase (Complex-IV) activity was evaluated according to the method described by Wharton [72]. The rate of oxidation of ferrocytochrome c is measured by following the decrease in the absorbance at 550 nm. Oxidized cytochrome c was reduced by adding potassium ascorbate. Then, 0.3 mM of reduced cytochrome c was added to 0.1 M potassium phosphate buffer (pH 7.0), 0.1 M potassium ferricyanide, and the reaction was initiated by mixing the appropriate amount of mitochondrial suspension. Results are expressed as nM cytochrome c oxidized /min/mg protein.

#### 4.6.2. Enzyme Activity Assay

Manganese superoxide dismutase (MnSOD) activity was measured by the method of Misra and Fridovich [73], based on the inhibition of autooxidation of adrenaline to adrenochrome by SOD contained in the examined samples. The samples were preincubated at 0 °C for 60 min with 6 mM KCN, which produces total inhibition of Cu, Zn-SOD activity. The results are expressed as specific activity of the enzyme in units per mg protein. One unit of SOD activity was defined as the amount of protein causing 50% inhibition of the conversion rate of adrenaline to adrenochrome under specified conditions.

The activity of selenium-dependent glutathione peroxidase (GPx) was determined according to the method of Flohé and Gunzler [74]. Briefly, the reaction mixtures consisted of 50 mM K_3_PO_4_ (pH 7.0), 1 mM EDTA, 1 mM NaN_3_, 0.2 mM NADPH, 1 mM GSH, 0.25 mM H_2_O_2_, and 226 U/mL glutathione reductase, and rates of NADPH oxidation followed at 340 nm.

Glutathione-S-transferase (GST) activity was determined by assaying 1-chloro-2, 4-dinitrobenzene (CDNB) conjugation with GSH, as described by Warholm et al. [75]. The working solution contained 20 mM CDNB, 20 mM GSH, and 1 mM EDTA in 200 mM phosphate buffer. The conjugated product was recorded at 340 nm continuously for 5 min at 30 °C (ε = 9.61 × 10^3^ M^−1^cm^−1^).

#### 4.6.3. Measurement of the Reduced and Oxidized Glutathione Contents

Total glutathione—the sum of reduced glutathione and oxidized glutathione (GSH and GSSG)—was determined by a method where glutathione is extracted from the mitochondria with 5% ice-cold sulfosalicytic acid and after neutralization with triethanolamine sequentially oxidized by DTNB (0.6 mM) and reduced by NADPH (0.3 mM) in the presence of glutathione reductase (2 U/mL) [76]. For determination of the GSSG alone, the GSH presented in solutions was derivatized by incubation with 2 µL 2-vinilpyridine at 4 °C for 1 h. The rate of 2-nitro-5-thiobenzoic acid formation was monitored at 412 nm and compared to standard curves made with GSH and GSSG, respectively. The GSH concentration is calculated as total glutathione—2 × [GSSG].

### 4.7. Western Blot Analysis

For the immunoblotting analysis, the isolated mitochondria were incubated with RIPA buffer containing 50 mM Tris-HCl pH 8.0, 150 mM NaCl, 1.0% Nonidet P-40, 0.5% sodium deoxycholate, 0.1% sodium dodecyl sulphate, 1mM phenylmethylsulfonyl fluoride, and 1 μg/mL Protease and Phosphatase inhibitor Cocktail (78440, Thermo Scientific Inc., Rockford, IL 61105, USA). The lysate was centrifuged at 14,000× *g* for 15 min. This fraction was labeled as the mitochondrial fraction and kept at −80 °C. The cytosol fraction was performed as follows. The brain and skeletal muscle tissues were homogenized in ice-cold lysis buffer containing 10 mM HEPES (pH 7.9), 1.5 mM MgCl_2_, 10 mM KCl, 1 mM dithiothreitol, 0.1 mM EDTA, and 0.2 mM phenylmethylsulfonyl fluoride plus 1 μg/mL Protease and Phosphatase inhibitor Cocktail (78440, Thermo Scientific Inc., Rockford, IL 61105, USA). These suspensions were incubated on ice for 15 min. Then, 12.5 μL of 10% Nonidet P-40 was added and the mixture was vigorously vortexed for 15 s. The cytoplasmic and nuclear fractions were separated by centrifugation at 15,000× *g* at 4 °C for 2 min. Then nuclei were resuspended in ice-cold high-salt extraction buffer (50 mM HEPES (pH7.8), 50 mM KCl, 300 mM NaCL, 0.1 mM EDTA, 0.1 mM phenylmethylsulfonyl fluoride, 1mM DTT, 1 μg/mL Protease and Phosphatase inhibitor Cocktail (78440, Thermo Fisher Scientific Inc., Rockford, IL 61105, USA), 10% (*v/v*) glycerol). The nuclear suspension was placed on a rocking platform for 30 min at 4 °C to facilitate lysis of the nuclei. The nuclear lysates were then centrifuged at 16,000× *g* for 10 min at 4 °C. The supernatant was collected and stored at −80 °C. Equal amounts of protein (100 μg) were mixed with Laemmli buffer (S3401, Sigma-Aldrich Inc., St. Louis, MO 63103, USA), heated (99 °C, 5 min), and then loaded onto 10–12% SDS polyacrylamide gels. Separated proteins were transferred onto polyvinylidene difluoride (PVDF) membranes, which were blocked in 5% non-fat milk in Tris-buffered saline-Tween (TBS-T) for 1 hour in room temperature. Primary antibodies were applied overnight at 4 °C. After washing in 1% non-fat milk in TBS-T (3 × 10 min) the membranes were incubated with a secondary antibody conjugated to horseradish peroxidase (HRP) for 1 h in room temperature. Each antigen–antibody complex was visualized by the amino-ethylcarbazol reaction. The relative expression of the proteins was quantified using densitometric scanning and analyzed by ImageJ and expressed as a percentage of controls. All samples were analyzed at least twice, and the average value was calculated for each sample. β-Actin and Lamin B1 were used as loading controls. Antibodies and dilutions were as follow: Nrf2 1:1000 (Sigma-Aldrich Inc., St. Louis, MO 63103, USA), MnSOD 1:500 (Sigma-Aldrich Inc., St. Louis, MO 63103, USA), p53 1:500 (Thermo Scientific Inc., Rockford, IL 61105, USA); Bcl-2 1:500 (Santa Cruz Biotechnology Inc., 69115 Heidelberg, Germany); GSTP 1:500 (Santa Cruz Biotechnology Inc., 69115 Heidelberg, Germany), GCLC 1:500 (Sigma-Aldrich Inc., St. Louis, MO 63103, USA), β-actin 1:1000 (Santa Cruz Biotechnology Inc., 69115 Heidelberg, Germany), Lamin B1 1:1000 (Santa Cruz Biotechnology Inc., 69115 Heidelberg, Germany), anti-mouse IgG HRP 1:1000 (Sigma-Aldrich Inc., St. Louis, MO 63103, USA), and anti-rabbit IgG HRP 1:1000 (Sigma-Aldrich Inc., St. Louis, MO 63103, USA). 

### 4.8. Statistical Analysis

Data are expressed as the mean ± standard deviation (SD) for each group. The differences among multiple experimental groups were detected by one-way Analysis of Variance (ANOVA) followed by Bonferroni’s multiple comparison test. The correlation between pairs of variables was analyzed using the bivariate Pearson method. A *p* value of less than 0.05 was considered significant.

## 5. Conclusions

Our results revealed that C_60_ prevented mitochondrial oxidative stress and mitochondrial dysfunction induced by a high dose of 3-NPA administration in rats. Beneficial effects of C_60_ were achieved, at least in part, by the elimination of ROS overproduction with the consequent decrease in LPO intensity as well as by the reduction of impairment of respiratory chain enzymes, especially SDH. Moreover, C_60_ modulated the pro/antioxidant balance and GSH/GSSG ratio in mitochondria. In addition to its strong antioxidant properties, C_60_ application inhibited the mitochondria-dependent apoptosis through the termination of p53 accumulation in mitochondria and the rise of Bcl-2 protein expression. Based on our studies, it could be concluded that the above effects and the degree of their manifestation depended on tissue specificity and C_60_ treatment regime. In brain mitochondria, C_60_ applied in the pre-treatment mode caused a significant decrease in oxidative stress markers and strengthening of the antioxidant capacity in comparison to the post-treatment regimen. It is possible to conclude that C_60_ preventive application was more effective than C_60_ injection after the oxidative intervention. At the same time, in skeletal muscle mitochondria, the intensification in antioxidant capacity was registered for the post-treatment regimen of C_60_ administration.

Similar changes in nuclear Nrf2 protein content and its targeted GSH-related enzymes permit us to suppose that under 3-NPA intoxication, along with other mechanisms, Nrf2/ARE-antioxidant pathway may be involved in the regulation of GSH homeostasis. Since exogenous injection of GSH is not effective, the modulation of glutathione biosynthesis by C_60_ could be an excellent therapeutic tool for the prevention or cure of neurodegenerative disorders caused by oxidative stress. However, further studies in relevant models would be necessary to provide new insights into the intrinsic molecular mechanisms of C_60_ protective effects. 

## Figures and Tables

**Table 1:** Effect of C_60_ pre- and post-treatment on movement analysis in rats subjected to 3-NPA.

Treatment	Number of Animals with Normal Behavior/Total Number of Animals Used (0)	Number of Animals with General Slowness/Total Number of Animals Used (1)	Number of Animals with Incoordination and Marked Gait Abnormalities/Total Number of Animals Used (2)	Number of Animals with Hind Limb Paralysis/Total Number of Animals Used (3)	Number of Animals with Incapacity to Move/Total Number of Animals Used (4)
Control	10/10	0/10	0/10	0/10	0/10
3-NPA	0/10	0/10	4/10	3/10	3/10
Pre-T	8/10	2/10	0/10	0/10	0/10
Post-T	6/10	3/10	1/10	0/10	0/10

**Table 2:** Experimental protocol used for 3-NPA and C_60_ fullerene treatment.

Groups	Days									
	−2	−1	0	1	2	3	4	5	6	7
3-NPA (alone)			●	●	●					
C_60_ fullerene pre-treatment	Δ	Δ	● Δ	● Δ	● Δ					
C_60_ fullerene post-treatment			●	●	●	Δ	Δ	Δ	Δ	Δ
C_60_ fullerene (alone)			Δ	Δ	Δ	Δ	Δ			

●—3-NPA treatment; Δ—C_60_ fullerene treatment.

## Data Availability

The data used to support the findings of this study are available from the corresponding author upon request.
